# Explainable Machine Learning for Public Health Informatics in HEDIS Childhood Immunization Status Combination 10

**DOI:** 10.2196/90241

**Published:** 2026-07-31

**Authors:** JiaLi Ruan

**Affiliations:** 1 Health Care Service Corporation, 1100 E Warrenville Rd, Naperville, IL, 60563, United States, 1 630-701-4247

**Keywords:** public health, childhood immunization, HEDIS, CIS Combo 10, National Immunization Survey, machine learning, random forest, explainable artificial intelligence, SHAP, public health informatics, quality measurement, Shapley Additive Explanations, Childhood Immunization Status Combination 10, Healthcare Effectiveness Data and Information Set

## Abstract

Completion of the HEDIS (Healthcare Effectiveness Data and Information Set) Childhood Immunization Status Combination 10 (Combo 10) measure among US children aged 24-35 months declined from 53.7% in 2021 to 44.6% in 2023, with a statistically significant survey-weighted annual trend. An explainable machine learning approach identified influenza vaccination and rotavirus series completion as the strongest component-level drivers of Combo 10 completion, supporting targeted public health quality improvement.

## Introduction

Immunizations have substantially reduced vaccine-preventable morbidity and mortality worldwide [1]. The Childhood Immunization Status (CIS) Combination 10 (Combo 10) measure is a core HEDIS (Healthcare Effectiveness Data and Information Set) pediatric quality indicator [2]. Combo 10 assesses whether children receive the complete series of 10 recommended vaccines by 24 months of age; however, national performance remains below desired benchmarks [3]. Identifying component-level drivers of Combo 10 failure is a public health informatics problem: it converts population-level quality data into actionable information for health plans, immunization programs, and policy stakeholders.

Traditional Combo 10 reporting summarizes overall completion but gives limited insight into component-level drivers. Public health informatics can extend routine quality reporting by linking population data, transparent analytics, and actionable interpretation. Explainable artificial intelligence (XAI) can show which inputs drive model output [4-9]. This study applied survey-weighted regression, random forest classification, and SHAP (Shapley Additive Explanations) to national survey data to identify components most predictive of HEDIS CIS Combo 10 completion.

## Methods

### Study Design

The 2021‐2023 National Immunization Survey–Child (NIS-Child) public-use files, a nationally representative CDC (Centers for Disease Control and Prevention) survey with provider-verified vaccination records, were analyzed. The age-eligible cohort included 32,997 children aged 24‐35 months, aligning with HEDIS CIS specifications. Weighted modeling was restricted to records with valid provider-adjusted child weights (PROVWT_C) and complete outcome information, yielding 16,021 children. Analytic features were 10 CDC-defined up-to-date (UTD) binary indicators corresponding to Combo 10 components: DTaP, IPV, MMR, Hib, hepatitis B, varicella, PCV, hepatitis A, rotavirus, and influenza. Indicators were harmonized across years; missing or insufficient provider records were coded as not UTD. Survey-weighted logistic regression estimated national trends, odds ratios (ORs), 95% CIs, and *P* values. For XAI analysis, a child-level random forest classifier modeled Combo 10 completion from the 10 UTD indicators, incorporating provider weights; 5-fold cross-validation was used as a model-checking step. Because Combo 10 is defined by these indicators, the classifier was used for SHAP-based decomposition rather than independent outcome prediction. SHAP values estimated each component’s contribution to Combo 10 classification; mean absolute SHAP values were used for global ranking.

### Ethical Considerations

This study used publicly available, deidentified NIS-Child public-use data and did not involve human subjects research as defined by federal regulations; therefore, institutional review board approval was not required.

## Results

**Figure 1.**
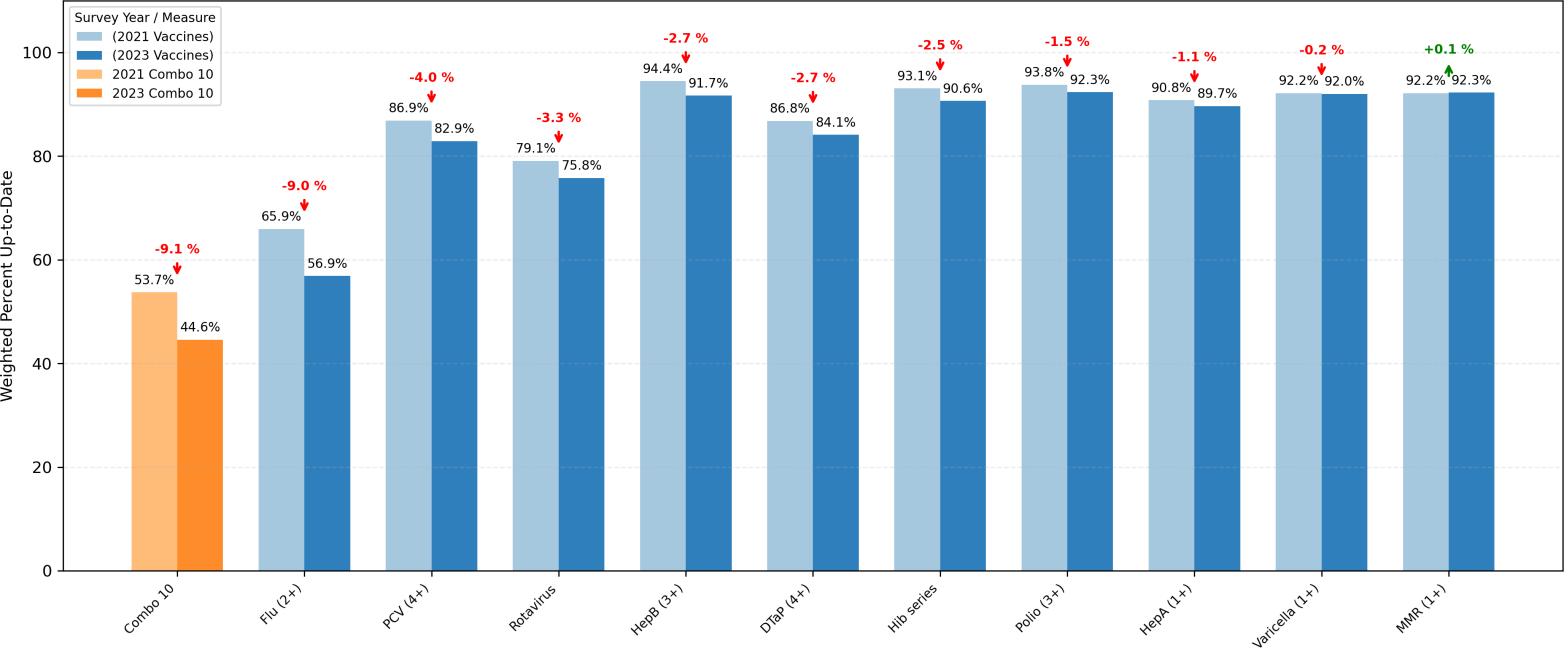
National vaccine coverage changes from 2021 to 2023, sorted by decline, with the Childhood Immunization Status (CIS) Combination 10 (Combo 10) measure highlighted.

The age-eligible cohort included 32,997 children; 16,021 had valid provider weights and complete outcome information. National HEDIS CIS Combo 10 completion declined from 53.7% in 2021 to 49.6% in 2022 and 44.6% in 2023, a 9.1-percentage-point decrease. The survey-weighted regression model was statistically significant for the annual trend (annual OR 0.83, 95% CI 0.831‐0.834; *P*<.001). Figure 1 shows nonuniform component declines, supporting component-level informatics analysis rather than aggregate rates alone.

**Figure 2.**
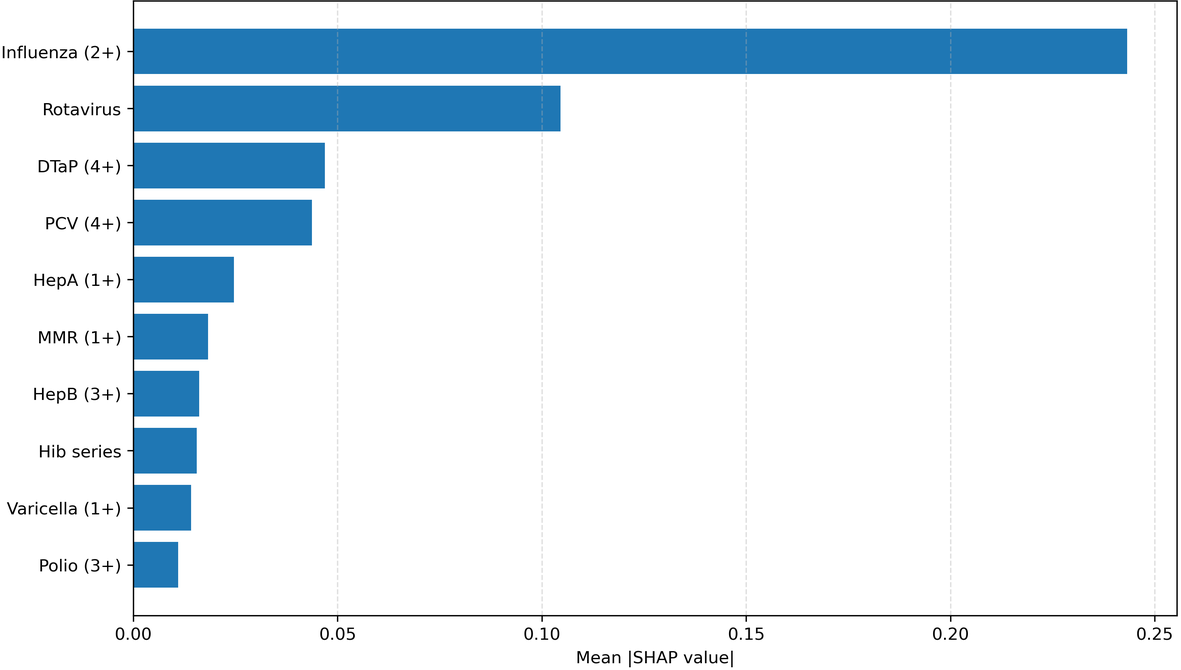
Mean absolute Shapley Additive Explanations (SHAP) values for CIS Combination 10 (Combo 10) prediction.

Five-fold cross-validation was used to check model stability. Because CIS Combo 10 completion is defined by the 10 vaccine component indicators, classifier performance was expected to be near-perfect and was not interpreted as independent predictive validity. The random forest model was used for SHAP-based decomposition. SHAP rankings showed that influenza vaccination and rotavirus series completion had the largest mean absolute SHAP values and were therefore the strongest drivers of predicted Combo 10 status. DTaP and PCV had moderate importance, while MMR, varicella, IPV, Hib, and hepatitis B had lower SHAP influence. Figure 2 displays SHAP-based feature importance rather than native impurity-based importance measures.

## Discussion

The main finding was that incomplete influenza and rotavirus vaccination disproportionately explained HEDIS CIS Combo 10 underperformance in this national sample. This pattern is actionable because practices and health plans can contact patients who are due or overdue for immunizations, use visit-based prompts to reduce missed vaccines, and train staff to discuss immunization with families. Future motivational interviewing programs could pair these outreach workflows with family-centered communication and could be adapted to other HEDIS measures to improve public health quality.

This study illustrates how XAI can complement quality-measure reporting within public health informatics. Rather than treating machine learning as a black box, SHAP linked each prediction to vaccine-specific inputs and distinguished components merely included in HEDIS from those driving noncompletion. The approach can support transparent, data-driven quality improvement for clinicians, health plans, public health agencies, and policy stakeholders [5,6,8,9].

Limitations include use of public-use NIS-Child data rather than plan-level HEDIS claims and reliance on a global SHAP bar plot. SHAP values indicate model-based component contributions rather than causal effects; future public health informatics work should validate findings in plan-level HEDIS data and examine subgroup patterns with beeswarm or dependence plots.

## Abbreviations

CDC: Centers for Disease Control and Prevention
CIS: Childhood Immunization Status
Combo 10: Combination 10
HEDIS: Healthcare Effectiveness Data and Information Set
NIS-Child: National Immunization Survey–Child
OR: odds ratio
SHAP: Shapley Additive Explanations
UTD: up to date
XAI: explainable artificial intelligence
